# The Value of Somatostatin Receptor Imaging with In-111 Octreotide and/or Ga-68 DOTATATE in Localizing Ectopic ACTH Producing Tumors

**DOI:** 10.4274/Mirt.69775

**Published:** 2013-08-01

**Authors:** Zeynep Gözde Özkan, Serkan Kuyumcu, Deniz Balköse, Berker Özkan, Nihat Aksakal, Ebru Yılmaz, Yasemin Şanlı, Cüneyt Türkmen, Ferihan Aral, Işık Adalet

**Affiliations:** 1 İstanbul University İstanbul Medical Faculty, Nuclear Medicine Department, İstanbul, Turkey; 2 Mehmet Akif Ersoy Thoracic and Cardiovascular Surgery Research and Education Hospital, Nuclear Medicine Department, İstanbul, Turkey; 3 İstanbul University İstanbul Medical Faculty, Thoracic Surgery Department, İstanbul, Turkey; 4 İstanbul University İstanbul Medical Faculty, General Surgery Department, İstanbul, Turkey; 5 İstanbul University İstanbul Medical Faculty, Endocrinology and Metabolism Department, İstanbul, Turkey

**Keywords:** Cushing syndrome, ectopic ACTH Syndrome, indium-111-octreotide, somatostatin receptor, scintigraphy, gallium radioisotopes, positron-emission tomography/computed tomography

## Abstract

**Objective:** We aimed to evaluate the value of somatostatin receptor imaging (SRI) with In-111 octreotide and Ga-68 DOTATATE in localizing ectopic ACTH producing tumors.

**Methods:** Nineteen patients who had In-111 octreotide somatostatin receptor scintigraphy (SRS) and/or Ga-68 DOTATATE PET-CT to localize ectopic ACTH producing tumors between the years 2000 and 2012 were included retrospectively in our study. The results of SRI were compared with clinical onset, radiological findings and surgical data of the patients.

**Results:** Sixteen In-111 octreotide SRS and five Ga-68 DOTATATE PET-CT were performed in 19 patients. In eight out of 19 patients, ectopic ACTH secretion site could be detected. In five patients, SRS showed pathologic uptake. In four of these patients, surgery revealed pulmonary carcinoid tumors and in one patient pancreatic neuroendocrine tumor. In one patient, Ga-68 DOTATATE PET-CT revealed pathologic uptake in lung nodule which came out to be pulmonary carcinoid tumor. In another patient who had resection of metastases of atypical carcinoid tumor prior to scans, new metastatic foci were detected both with SRS and Ga-68 DOTATATE PET-CT imaging. In one patient, although SRS was negative, CT which was performed three years later showed a lung nodule diagnosed as pulmonary carcinoid tumor. In 11 patients, ectopic ACTH secretion site could not be detected. In 10 of those patients, scintigraphic and radiological imaging did not show any lesions and in one patient, Ga-68 DOTATATE PET-CT was false positive.

**Conclusion:** SRI has a complementary role with radiological imaging in localizing ectopic ACTH secretion sites. PET-CT imaging with Ga-68 peptide conjugates is a promising new modality for this indication.

**Conflict of interest:**None declared.

## INTRODUCTION

Hypersecretion of hormones from adrenal cortex causes a complex cascade of effects which is called Cushing’s syndrome. Most of the abnormalities of Cushing’s syndrome are caused by abnormal amounts of cortisol (1). Cushing’s syndrome is classified into 2 types as ACTH-dependent and ACTH-independent. Excess amount of cortisol is the result of primary adrenal overproduction in the ACTH-independent type and ACTH levels are low or in undetectable amounts. In the ACTH-dependent type, there is overproduction of ACTH either from pituitary adenoma (Cushing’s disease) or hypothalamic-pituitary dysfunction or ectopic secretion from tumors (1). Ectopic ACTH secretion accounts for approximately 15-20% of ACTH-dependant Cushing’s syndrome (2). 

Localization of ACTH secreting tumors is a challenging process. Ilias et al reported that in 19% of their patients, a tumor could not be identified despite extensive evaluation (3). Hernandez et al managed to localize the source of ectopic ACTH secretion in 62.5% of their patients by using multiple imaging modalities (4). Although there are controversial results about its performance in the literature, somatostatin receptor scintigraphy (SRS) with In-111 octreotide had been used to detect ectopic ACTH secreting tumors (2,3,4,5,6,7,8,9,10,11,12). Ga-68 peptide conjugates had begun to be used recently for the similar indications with In-111 octreotide. There are several case reports about detection of ectopic ACTH secreting tumors with Ga-68 peptide conjugates (13,14,15).

In this study, the value of somatostatin receptor imaging (SRI) with In-111 octreotide and Ga-68 DOTATATE in detection of ectopic ACTH secretion was evaluated. 

## MATERIALS AND METHODS

**Patients**

Twenty-nine patients with suspicion of ectopic ACTH syndrome who were referred to our department for SRS with In-111 octreotide between 2000 and 2011 (24 patients, 29 scans) and for Ga-68 DOTATATE PET-CT between 2011 and 2012 (8 patients, 9 scans) were retrospectively evaluated. Informed consent of every patient was obtained prior to imaging. Ten patients were excluded from the study due to pituitary adenomas which were detected with cranial MRI during follow-up. SRS with In-111 octreotide in 16 patients and Ga-68 DOTATATE PET-CT scans in five patients were performed in a total of 19 patients. In two patients, both In-111 SRS and Ga-68 DOTATATE PET-CT were done.

There were 13 female and six male patients. The ages ranged between 14 and 61 years. The mean age was 37,8 years. Two patients had pituitary surgery, three patients had bilateral adrenalectomy, one patient had pituitary surgery and bilateral adrenalectomy, one patient had mediastinal mass resection before In-111 octreotide SRS or Ga-68 DOTATATE PET-CT was performed.

**Patient Preparation**

For patients who were under somatostatin therapy, In-111 octreotide SRS or Ga-68 DOTATATE PET-CT was performed four weeks after the therapeutic injection of somatostatin. The patients were asked to use laxatives for bowel cleaning and to eat a light diet before In-111 octreotide administration and during imaging period. Oral contrast agent was given to patients for Ga-68 DOTATATE PET-CT.

**Somatostatin Receptor Scintigraphy**

Five mCi of In-111 octreotide was administered to patients intravenously. Imaging was performed by either Siemens ECAM or ADAC Vertex Plus Gama-camera. Whole body image acquisition was performed in anterior and posterior positions (10 cm/min) four hours after injection. Subsequent anterior and posterior static images (106 counts) of the thorax and abdomen were also performed. Late static images of thorax and abdomen were acquired 24 hours after injecton as well as SPECT image acquisition. SPECT images were acquired according to the following parameters: 64 frames, 128x128 matrix, 360° rotation, 40 seconds/frame. Image acquisition was also performed at 48 hours when necessary. CT of the appropriate regions were also performed on spiral 4 slice CT (CT component of Biograph™ TruePoint™ PET-CT) with a slice thickness of 4 mm in 7 patients. CT images and reconstructed SPECT images were evaluated after fusion images were rendered using Siemens SYNGO™ software.

**Ga-68 DOTATATE PET-CT Imaging**

Ga-68 DOTATATE was prepared in-house on a fully automated system (Eckert & Ziegler Eurotope, Berlin, Germany). The images were obtained on a dedicated PET/CT scanner (Biograph™ TruePoint™ PET/CT), 60 min after intravenous injection of 3-4 mCi of Ga-68 DOTATATE . CT acquisition was performed on spiral four slice CT with a slice thickness of 4 mm. After transmission scan, 3D PET acquisition was taken for 4 min per bed position for 6-8 bed positions. CT-based attenuation correction of the emission images was employed. PET images were reconstructed by iterative method ordered subset expectation maximization (2 iterations and 8 subsets) with filter size of 5 mm. After completion of PET acquisition, the reconstructed attenuation corrected PET images, CT images and fused images of matching pairs of PET and CT images were available for review in axial, coronal, and sagittal planes and in maximum intensity projections three dimensional cine mode.

**Evaluating SRS and PET-CT Images**

A positive scan was defined as significant accumulation of tracer, based on visual assessment. The images were reviewed for areas of abnormally increased tracer uptake other than physiological uptakes in thyroid, spleen, liver, kidneys, pituitary gland, bowel and bladder by an experienced nuclear medicine physician. For SRS images, abnormal uptakes on the early static images were followed on the late static images, which were acquired at 24 hours and if the abnormal uptake remained or its intensity was increased, it was considered as pathological.

**Patient Evaluation**

The results of In-111 octreotide SRS and Ga-68 DOTATATE PET-CT were evaluated in comparison to clinical onset, radiological findings and postsurgical histopathological examination of the patients.

## RESULTS

**Figure 1:**
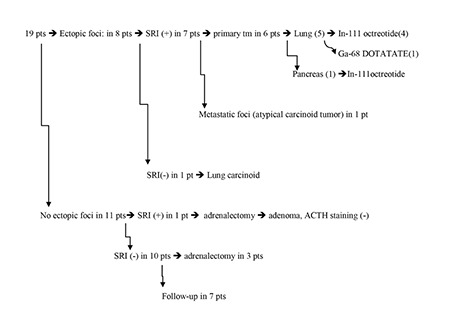
Summary of patient outcomesSRI: Somatostatin receptor imagingPt: patient

In eight out of 19 (42.1%) patients, ectopic ACTH secretion sites were detected. In six patients, pulmonary carcinoid tumor and in one patient pancreatic neuroendocrine tumor was the reason of ectopic ACTH secretion. In the last patient, metastatic foci of atypical carcinoid tumor of the unknown origin were the sites of ACTH secretion. The summary of patients’ outcome is given in Figure 1. In seven of these eight patients (87.5%), SRI were positive.

**Figure 2:**
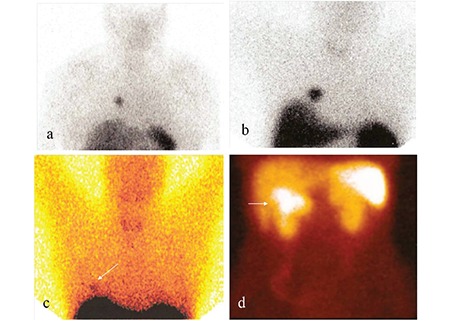
Planar images of In-111 octreotide SRS of 4 patients are shown: Pulmonary carcinoid tumors (A-C) and pancreatic neuroendocrine tumor (D) Please provide a better copy of Figure 2 C).

**Figure 3:**
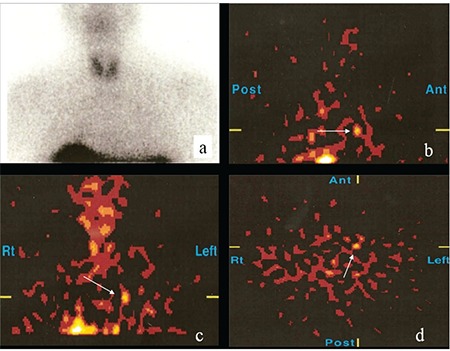
Planar and SPECT images of In-111 octreotide SRS of a patient with pulmonary carcinoid tumor. Although the planar image is negative (A), in sagittal (B), coronal (C) and axial (D) SPECT images the lesion can be seen

**Figure 4:**
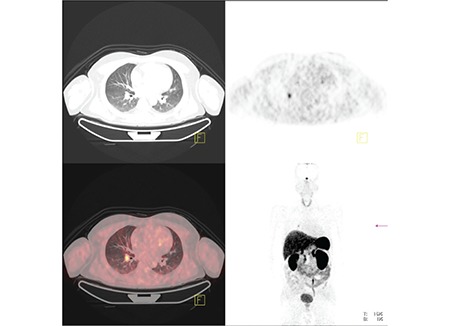
The centrally located pulmonary nodule in right lung lower lobe cannot be detected easily in CT image, but there is high Ga-68 DOTATATE uptake in PET image. The nodule can be easily detected in PET-CT fusion image.

In four patients with pulmonary carcinoid tumors, there were pathological In-111 octreotide uptakes in the tumors (Figure 2A-C). In one of these patients, there was no pathological In-111 octreotide uptake in the planar images, but only in SPECT images (Figure 3). Other 3 patients had In-111 octreotide uptake on both planar and SPECT images. In all of these patients, there were nodules which corresponded to these uptake sites on CT images performed before SRS. All patients underwent surgery and postsurgical histopathological examination revealed pulmonary carcinoid tumors. Size of the tumors ranged between 0.8 to 2 centimeters. In another patient with pulmonary carcinoid tumor, there was no pathological In-111 octreotide uptake on SRS. A follow-up CT which was peformed three years after SRS revealed a one centimeter nodule in left lung upper lobe. The nodule was resected and came out to be a carcinoid tumor. In the last patient with pulmonary carcinoid tumor, there was high Ga-68 DOTATATE accumulation in the nodule which was 1,3 cm in diameter, located centrally at right lung lower lobe (Figure 4). The CT images performed before Ga-68 DOTATATE PET-CT was evaluated as negative for this suspicious lesion. The patient had a right lung lower lobe resection and the pathology specimen confirmed an atypical carcinoid tumor. 

In the patient with pancreatic neuroendocrine tumor, there was a mass of 5 cm diameter detected in MR imaging prior to SRS, located between duodenum, pancreatic head and mesenteric vascular bed. There was pathological In-111 octreotide uptake in the tumor (Figure 2D). This patient underwent pancreatoduodenectomy and surgical resection material revealed a neuroendocrine tumor. 

The last patient with positive SRI was diagnosed with metastatic atypical carcinoid tumor following resection of mediastinal mass and lymph nodes. SRS was performed in order to localize other ectopic sources of ACTH since the clinical symptoms of the patient did not improve in the postsurgical period. Planar and SPECT-CT images of SRS demonstrated faint uptake of In-111 octreotide in metastatic lymph nodes and bone lesions. Patient underwent chemotherapy but her clinical status worsened in long term follow up. Ga-68 DOTATATE PET-CT imaging had showed progression of the disease.

**Figure 5:**
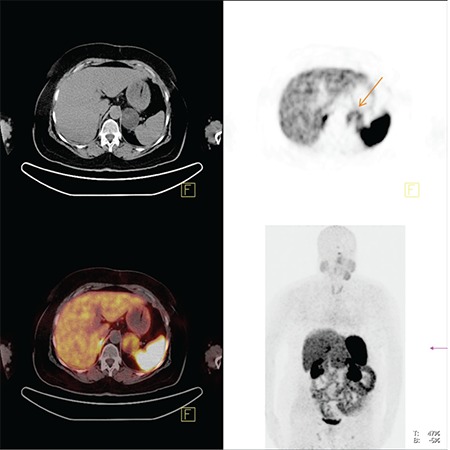
The moderate Ga-68 DOTATATE uptake in the left adrenal lesion led to false positive interpretation of the image as the site of ectopic ACTH secretion, but the pathology revealed adrenal adenoma without ACTH staining.

In 11 patients, ectopic ACTH secretion site could not be localized. In 10 of these patients there was neither any pathologic tracer uptake in SRI nor any lesions in radiologic imaging. In seven of these 10 patients, the clinical onset of the disease was mild and they were followed-up. In the remaining three patients, the severe clinical onset of the disease led to bilateral adrenalectomy. In the last patient, a mass was detected in left adrenal gland, 4 cm in diameter on CT images. There was a moderate Ga-68 DOTATATE uptake in this lesion (Figure 5). Although the ectopic ACTH secretion was thought to be from this lesion, the adrenalectomy material revealed an adenoma without ACTH staining. ACTH level of the patient did not decrease after the operation. Ga-68 DOTATATE PET-CT result is accepted to be false positive in this patient.

## DISCUSSION

Evaluating patients with suspected ectopic ACTH production is a challenging issue. The main problem is the localization of the ACTH secreting tumor. In our patient group, there were 19 patients with the diagnosis of ectopic Cushing’s syndrome, but only in eight patients (42.1%), the foci of the disease could be detected. In the literature there are varying results. Ilias et al reported that in 19% of their patients, a tumor could not be identified (3), whereas Hernandez et al could localize the tumors in 62.5% of their patients (4). 

There are controversial results about the performance of SRI in the literature. In our study, seven patients out of eight (%87.5) had positive SRI, either with In-111 octreotide or Ga-68 DOTATATE. Zemskova et al reported the sensitivity of In-111 octreotide SRS for detection of the ectopic ACTH source as 57%, whereas the positive predictive value was 79% (11). Ejaz et al reported a sensitivity of 60%, Loli et al 67%, Ilias et al. 49% (3,8,12). In the study of De Herder et al., SRS have succesfully visualized ectopic ACTH producing tumors in eight of the 10 patients (5). In another study by Tsagarakis et al., in eight of 11 patients, ectopic ACTH producing tumors were succesfully detected by SRS (7). On the other hand, Torpy et al reported that only six SRS of 18 patients were positive (6). Similarly, in the study of Tabarin et al., only four patients out of 12 had positive SRS (2). Ilias et al stated that SRS could not show any lesions not seen on CT or MR (3). Tabarin et al emphasized that conventional imaging such as CT and MR was more helpful than SRS (2). In another study, it was stated that SRS could not find otherwise unsuspected lesions (10). On the other hand, there are some articles which emphasized the high diagnostic yield of SRS (5,7). In our study, only in one patient CT was positive while SRS, which was done three years ago, was normal. SRS might be positive, if it was repeated. 

It should be kept in mind that detectability of lesions in scintigraphic imaging depends on various factors such as lesion size, location, type, degree of somatostatin receptor expression and the amount of radioactivity in the lesion (9). In lesions smaller than one centimeter like pulmonary carcinoid tumors, extra attention must be paid while evaluating SRS images. For instance, performing SPECT or SPECT/CT imaging may overcome resolution problems compared to planar images. In one of our patients, planar SRS images were negative, while on SPECT images, a pathological uptake was seen in lung nodule which was 0.8 cm in diameter. Tabarin et al also emphasized the importance of SPECT imaging (2). With the improvement of the resolution, small lesions can be detected and especially in the organs where background activity is high, more succesful identification of the lesions can be done. We think that performing a thorax SPECT/CT imaging in the patients who have no pathologic In-111 octreotide on whole body and planar images will enable to localize small pulmonary lesions most of which will be carcinoid tumors. 

It can be predicted that Ga-68 peptide imaging can also have succesful results in ectopic ACTH producing tumors, given the better resolution than In-111octreotide. In the recent years, there have been some efforts for imaging somatostatin bearing tumors with Ga-68 peptides. Several studies have reported succesful results with Ga-68 peptide imaging in neuroendocrine tumors and bronchial carcinoid tumors (16,17,18). In our study, tumor of one patient was identified by Ga-68 DOTATATE PET-CT. The thorax CT which was done before PET-CT could not localize the tumor. Loli et al stated that small bronchial carcinoids sometimes could not be detected or identified because of their inner location made them look like normal vessels (8). There are also several case reports about detection of ectopic ACTH secreting tumors with Ga-68 peptide conjugates (13,14,15). At this point, it should be remembered that our only false positive SRI was moderate uptake of Ga-68 DOTATATE in surrenal adenoma. More studies are needed to evaluate the efficacy of Ga-68 peptide conjugate imaging with PET-CT in localizing ectopic ACTH secreting tumors.

In our study, six (75%) of the localized tumors were pulmonary carcinoids. Tsagarakis pointed out that bronchial carcinoids were the most common cause of ectopic Cushing’s syndrome (7). Due to their small size, localizing these tumors are quite difficult and as stated by Torpy et al, they constitute the majority of occult ectopic ACTH secreting tumors (6). Whereas in the literature, multiple studies most of which were case reports demonstrated ACTH producing bronchial carcinoid tumors using different radiopharmaceuticals such as In-111 octreotide, Tc 99m-ocreotide, F-18 FDG and Ga-68 DOTATATE (3,4,5,7,8,9,10,12,14,19,20,21,22,23,24,25,26,27,28,29,30). Small cell lung carcinomas may also produce ACTH (5,9,10,12). Therefore, while examining images for ectopic ACTH producing tumors, extra attention has to be paid to thoracic regions. 

## CONCLUSION

The localization of ectopic ACTH secreting tumors is a challenge. In-111 octreotide SRS have been used together with radiological modalities for a longtime. Ga-68 peptide conjugate imaging with PET-CT is a new promising modality for this indication. The pulmonary carcinoid tumors are responsible for the ectopic ACTH secretion in most of the cases. Therefore thoracic regions must be evaluated with great care in both In-111 octreotide SRS and PET-CT with Ga-68 peptide conjugates.
